# Replication-dependent histone isoforms: a new source of complexity in chromatin structure and function

**DOI:** 10.1093/nar/gky768

**Published:** 2018-08-27

**Authors:** Rajbir Singh, Emily Bassett, Arnab Chakravarti, Mark R Parthun

**Affiliations:** 1 Department of Radiation Oncology, The Ohio State University, Columbus, OH 43210, USA; 2 Department of Biological Chemistry and Pharmacology, The Ohio State University, Columbus, OH 43210, USA

## Abstract

Replication-dependent histones are expressed in a cell cycle regulated manner and supply the histones necessary to support DNA replication. In mammals, the replication-dependent histones are encoded by a family of genes that are located in several clusters. In humans, these include 16 genes for histone H2A, 22 genes for histone H2B, 14 genes for histone H3, 14 genes for histone H4 and 6 genes for histone H1. While the proteins encoded by these genes are highly similar, they are not identical. For many years, these genes were thought to encode functionally equivalent histone proteins. However, several lines of evidence have emerged that suggest that the replication-dependent histone genes can have specific functions and may constitute a novel layer of chromatin regulation. This Survey and Summary reviews the literature on replication-dependent histone isoforms and discusses potential mechanisms by which the small variations in primary sequence between the isoforms can alter chromatin function. In addition, we summarize the wealth of data implicating altered regulation of histone isoform expression in cancer.

## INTRODUCTION

Nucleosomes are highly dynamic structures, comprising 146 base pairs of DNA wrapped about 1.7 turns around an octameric histone core in a left-hand orientation. This core integrates two copies each of histones H2A, H2B, H3 and H4, which provide the fundamental architecture of the nucleosome. The core histones are arranged in a central (H3-H4)_2_ tetramer that is flanked on either side by two H2A–H2B dimers, and further stabilized by linker histone H1 (1–3). Overall, this interaction is very stable under physiological conditions, and is supported by 14 contact points between histones and DNA (2,4,5). This ‘beads on a string’ configuration is finally assembled into higher order tertiary structure known as chromatin, which after several orders of packaging and condensation results in the packaging of an entire chromosome. Nucleosome structure is vital for regulation of gene expression and many other DNA-dependent processes including transcription, replication, recombination, and repair. Any changes in nucleosome structure can affect the transcription kinetics and binding of various regulatory factors. Also, the nucleosomes are positionally malleable and can fully or partially disassemble based on the cellular milieu (6). All these factors govern the open and condensed chromatin states, which can modulate the trans-acting factors access to *cis*-acting regulatory elements (7,8). Specifically, three known mechanisms modulate nucleosome structure and chromatin dependent cellular processes:

### Histone modifications

The core histones have a globular domain and protruding NH_2_-terminal tails, both of which are subject to several post-translational modifications (2,3,9). The core histone tail domains are required for the assembly of higher-order structures and affect histone interactions with non-histone proteins as well as histone-histone and histone-DNA interactions (3,8,10,11). These modifications, including acetylation, ubiquitination, phosphorylation, methylation, sumoylation and ADP-ribosylation alter the structure and dynamics of the nucleosome, and accordingly, modulate chromosome function. Acetylation and phosphorylation can reduce the positive charge of the histones, thereby altering the electrostatic interactions and triggering structural change in histones or their binding to DNA. These modifications affect the compactness of chromatin structure, thereby governing DNA access by protein machineries (12–15). Similarly, histones can undergo monoubiquitination, which due to its large size can alter the overall conformation of the nucleosome, affecting intra-nucleosomal interactions as well as interactions with other chromatin-bound complexes. In contrast, neutral modifications like histone methylation may not manipulate chromatin structure, but may provide binding sites for several protein recognition modules. These domains include, but are not limited to, chromodomains, PHD fingers, Tudor, PWWP and MBT domains (12,14). Intriguingly, a cross-talk may also exist between different modifications, which may synergistically fine-tune the overall control, trans-regulating the nucleosomal interactions or binding of specific domains or effector molecules (16). So far, there are over 60 residues on histones that are known to be modified and eight distinct types of modifications (11,14). Given that there could be multiple modifications at the same site, and different types and sites of modification, a given nucleosome could exist in many thousands of combinatorial variations, each with a defined functional significance.

### Chromatin remodeling complexes

Several chromatin-remodeling enzymes facilitate the establishment of specific chromatin architecture during the course of any normal biological process. These enzymes mediate rapid rearrangements of chromatin structure that allows an access of condensed genomic DNA to the regulatory transcription machinery proteins, and thereby control gene expression. This dynamic modification regulates transcription, chromosome segregation, apoptosis, DNA replication, and DNA repair (17,18). Abnormalities in chromatin remodeling proteins are found to be associated with several human diseases and therefore chromatin remodeling pathways have been targeted for their therapeutic potential (19–21).

### Histone variants

It has been well established that the histone complement as such is not homogeneous. Nucleosomes also contain specialized variants that may replace the canonical histones in a context-dependent manner. Histone variants can be distinguished on several grounds. Conventionally, the variants may be defined as the nonallelic fraction of major histones that are replication independent and constitutively expressed throughout the cell cycle. They may differ from their canonical counterparts by a handful of amino acid changes to the incorporation of large non-histone domains. Additionally, histones are intronless, and their mRNAs are unique in terms of the presence of a unique stem–loop at the 3′-end. Histone mRNAs carry a six-base stem and four nucleotide loop at their 3′-end, unlike the poly (A) tail for other mRNAs (22). However, the histone variants may contain introns and they generate transcripts with normal polyadenylated tails. Lastly, the variants are found as single genes dispersed throughout the genome, and unlike the canonical histones, can show a large variation among species (23–25). The primary function of variants is not limited to packaging the DNA. Their array of functions includes, but is not limited to, the regulation of silencing, antisilencing, DNA repair and nucleosome stability. Histone variants are an important contributor to the structural and functional heterogeneity that is essential for the proper regulation of chromatin. This is evident from the presence of specific replacement variants that, unlike canonical histones, are expressed throughout the cell cycle and can supply the chromatin components needed during repair, recombination, and to replace histones lost through turnover in quiescent cells (26). Some well-characterized examples of replication-independent histone variants are histones H3.3, H2A.X, CenH3, H2A.Z and macroH2A (24,27–31). Their distribution pattern is contingent upon many factors including epigenetic changes and heterochromatin structure. For example, CenH3 is largely centromeric, which is imperative for its structural and functional roles (32). The nonhistone domain containing variant, macroH2A, is enriched on the transcriptionally inactivated female X chromosome, senescence-associated heterochromatic foci (SAHF), and other transcriptionally silent domains. Overall, many of these variants function as an ensemble, and any alterations in their localization and distribution can affect the higher-order chromatin organization, compromising mitotic progression and genome stability (32,33).

## DISCUSSION

Our definition of variants has been restricted to histones that meet the above-mentioned criteria. In this review, we focus on another category of variants that lies within the replication-dependent histone gene clusters and which are typically thought of as being functionally equivalent. We refer to this particular subset of variants as ‘isoforms’ to distinguish them from the replication-independent histone variants. Here, we examine the different human histone isoforms and highlight their functional role as epigenetic regulators. We also provide an overview of the existing literature about these isoforms and discuss their potential role in carcinogenesis.

### An introduction to histone isoforms

Canonical human histones are multigene families found at four loci, encompassing a total of 72 histone genes. The major histone gene cluster is located on chromosome 6 (HIST1 locus), which encodes a total of 55 histone genes (80% of the total fraction). Histone clusters 2, 3 and 4 (HIST2, HIST3 and HIST4 loci) are mapped to chromosome 1 and encode 12, 4 and 1 histone genes, respectively. Intriguingly, the HIST3 cluster may encode testis-specific histone proteins (34). The nomenclature for these histone isoforms consists of three parts. For each gene, the first part of the name refers to the histone cluster (i.e. HIST1, HIST2, HIST3 or HIST4). The second part of the name refers to the type of histone (i.e. H1, H2A, H2B, H3 or H4). Finally, each gene name has a letter where the first gene of each histone type is designated ‘A’ (telomere proximal) and subsequent genes of that histone type are designated in alphabetical order. For example, the fourth histone H2A gene in the HIST1 cluster would be named HIST1H2AD. Together, these 72 genes are known to encode five distinct histone subtypes, canonical histones H2A (16 genes), H2B (22 genes), H3 (14 genes), H4 (14 genes) and the linker histone H1 (6 genes). This redundancy, as it may appear, is not absolute. The products of many of these genes are not identical and differ by a few residues. Consequently, there is heterogeneity within the replication-dependent histone clusters, which may influence various biological processes and disease states.

**Figure 1.**
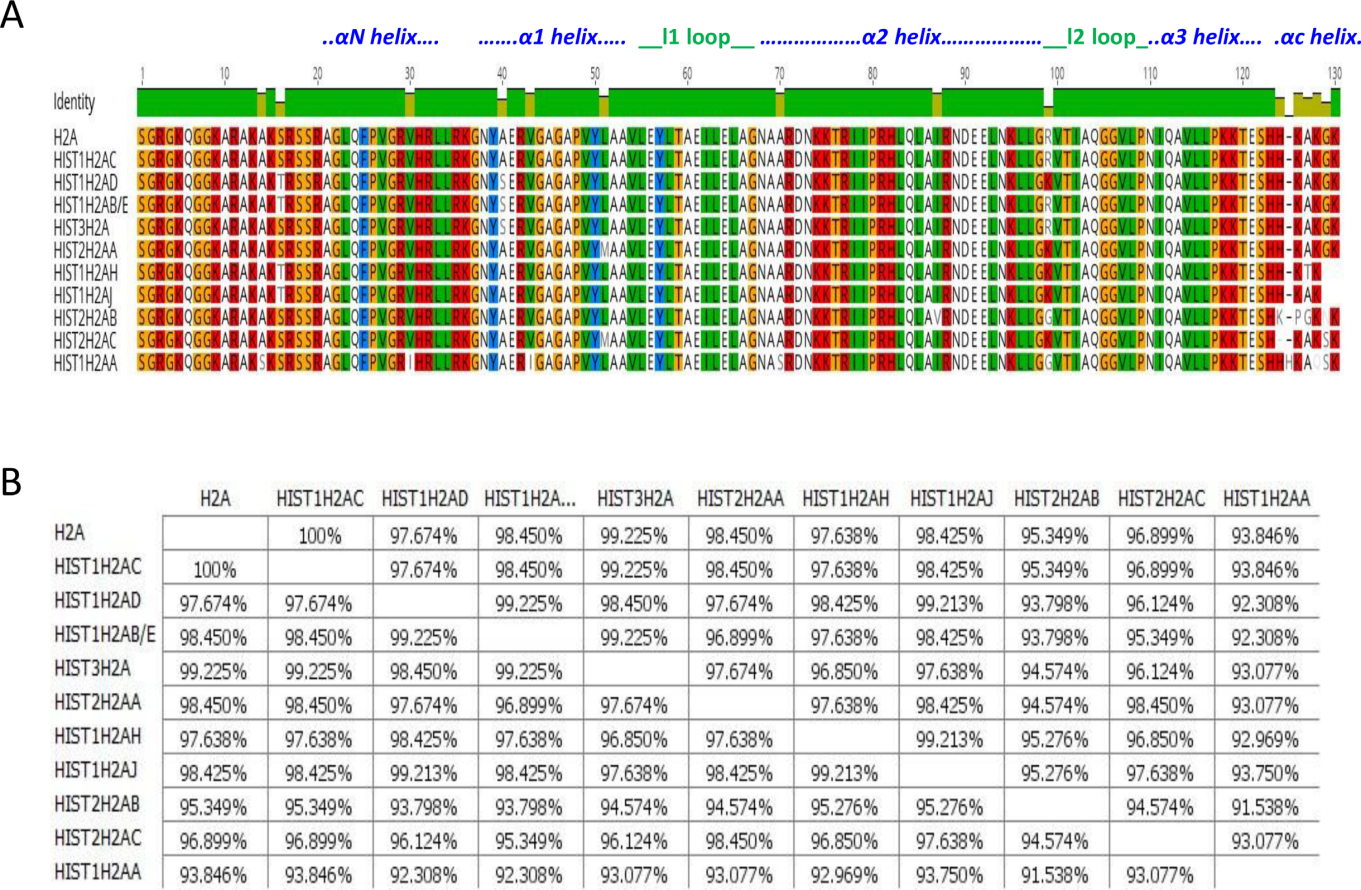
Comparison of H2A isoforms. (**A**) Sequence alignment of H2A isoforms. Residues that are different among the isoforms are shown. (**B**) Percentage similarity among the H2A isoforms based on their primary sequence. Images were created using the Geneious software.

**Figure 2.**
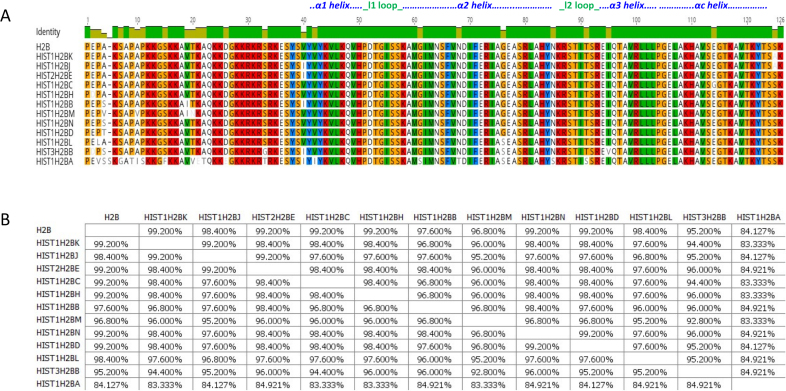
Comparison of H2B isoforms. (**A**) Sequence alignment of H2B isoforms. Residues that are different among the isoforms are shown. (**B**) Percentage similarity among the H2B isoforms based on their primary sequence. Images were created using the Geneious software.

**Figure 3.**
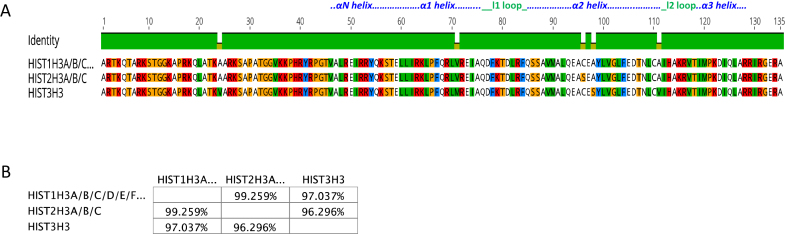
Comparison of H3 isoforms. ( **A**) Sequence alignment of H3 isoforms. Residues that are different among the isoforms are shown. (**B**) Percentage similarity among the H3 isoforms based on their primary sequence. Images were created using the Geneious software.

**Figure 4.**
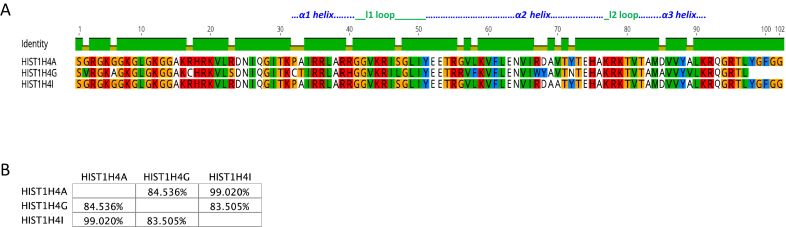
Comparison of H4 isoforms. ( **A**) Sequence alignment of H4 isoforms. Residues that are different among the isoforms are shown. (**B**) Percentage similarity among the H4 isoforms based on their primary sequence. Images were created using the Geneious software.

Figures 1 to 4 show sequence comparisons and the percentage identity between the four core histone isoforms. While, the function of linker histone isoforms is also intriguing, they will not be discussed in this review (35–38). For reference, we will refer to the histone isoform that is encoded by the largest number of genes as the canonical form of that histone. It is clear that the core histones differ in the degree of diversity in their number of isoforms. H2A and H2B are encoded by 11 and 13 isoforms, respectively, while H3 and H4 each have only three isoforms. It is also evident that the histone isoforms are highly similar. All of the H2A isoforms are greater than 90% identical. Similarly, all of the H2B isoforms are >90% identical with the exception of HIST1H2BA, which is testis-specific, and has a more variable NH_2_-terminal tail sequence. The isoforms of H3 and H4 are also >90% identical except for HIST1H4G, which has a truncated C-terminus.

Location within the gene clusters may have some influence on isoform sequence. For example, examination of the C to S variation at position 96 indicates that there is a C in all the human HIST1H3 genes and an S in the HIST2 cluster genes. Likewise, for the H2A genes, an L is found at position 51 in the isoforms in the HIST1 cluster while an M is at this position is only observed in isoforms in the HIST2 cluster. The factors that regulate the histone isoform expression within the clusters are far from clear. While genetic drift may contribute to some of the heterogeneity at the protein level, other sources of divergence may include a differential regulation, and the evolutionary favoring the selection of specific isoforms (26).

Despite their high degree of similarity, the subtle differences between isoforms may be significant as small changes in amino acid sequence may influence nucleosome structure or interactions with non-histone proteins. Therefore, the presence of distinct replication-dependent histone isoforms in the genome, may expand the complexity of mammalian chromatin structure. Given the fact that chromatin structure and dynamics are altered in many disease states, these particular isoforms could have a distinct role in human pathogenesis.

### Regulation of individual histone isoform expression

In order to meet the high demand of histones required for packaging the newly synthesized DNA, the expression of canonical histones is prominently induced during the S-phase of the cell cycle (22). Furthermore, histone gene expression is controlled at both transcriptional and post-transcriptional levels by the regulation of nuclear RNA processing, translation, and mRNA stability (39). Given the clustering and colocalization of the histone genes, it seems plausible to hypothesize that histone genes are transcriptionally regulated as a group. In this scenario, the histone isoforms should have similar gene expression levels and should be subject to common gene regulatory mechanisms. However, recent studies suggest that not only the expression of histone isoforms differs to a high degree, but their expression is also cell-type and context-dependent (40,41). This was supported by several studies that highlighted differential histone isoform expression (42–45). In addition, discrete sequences in the 5′-UTR of Hist1H2AC gene play a critical role in its posttranscriptional regulation (41). Two highly similar 15/16 base sequences (UGAUUUUGUUUGUUU and UGAUUUUUGUCUGAUU), span bases 28–42 and 71–86 of the HIST1H2AC 5′ UTR. These sequence elements were found to contain poly U stretches, and played a critical role in the posttranscriptional regulation of the HIST1H2AC gene (41). Another study highlighted that only the oligouridylated form of *Hist1h2ak* is a target for Eri1-dependent degradation, suggesting that oligouridylation interferes with histone mRNA stability and protein expression (46). These examples substantiate the idea that histone mRNAs are subject to a specific regulation.

The histone isoforms also show distinct patterns in the epigenetic regulation of their expression. In a genome-wide DNA methylation profile, Hist3H2A was found to be hypermethylated, specifically in the cancer stem cell enriched side population (SP) cells of Hepatocellular Carcinoma (HCC) (47). A study by Jung *et al.* analyzed the correlation of DNA methylation markers with overall survival of HCC patients. HIST1H2AE was one of the prominent CpG loci that exhibited an association between gene hypermethylation and poor prognosis. Multivariate analysis using the COX proportional hazards model demonstrated that the patients with methylated Hist1H2AE were at >2.5-fold increased risk for HCC, when clinicopathological factors including tumor size, serum GGT levels, and microscopic vascular invasion were taken into consideration (48). Similarly, a genetic-and-epigenetic cell cycle networks study by Li *et al.* highlighted the role of methylation of HIST1H2AJ in HeLa cells leading to an increase in the rate of cell proliferation and anti-apoptosis through modulating NF-κB, TGF-β and PI3K pathways (49). HIST1H4L was also found to be hypermethylated in hepatocellular carcinoma tissues and triple negative breast cancer cells (50–52). Hypermethylation of specific histone isoforms genes have also been discussed in multiple other reports including Hist1H2BK in breast cancer and constitutive methylation of HIST1H2AA in a Phase I trial of azacytidine treatment for resectable gastric and esophageal adenocarcinoma patients (53,54). Therefore, these results suggest that histone isoforms are subject to individualized regulation at transcriptional and posttranscriptional levels and may have distinct cellular functions.

### Functional relevance of histone isoforms

Despite these intriguing observations, very few studies have directly addressed the functional significance of histone isoforms. Recent reports have provided support for the hypothesis that histone isoforms can be functionally distinct proteins (40,41). Using mass spectrometry, changes in the complement of H2A proteins between normal and tumor tissue samples were observed, including a reduction in the levels of the H2A isoform encoded by the HIST1H2AC locus. RNAi-mediated reductions in the expression of H2A isoforms demonstrated that HIST1H2AC expression specifically influenced cell proliferation and tumorigenicity, *in vitro* (40,41,43).

A similar study by Monteiro *et al.* employed mass spectrometry to screen HC11 mammary epithelial cells for changes in histone levels throughout cell differentiation (43). They observed an increased abundance of Hist2H2AC protein specifically in undifferentiated/proliferating cells, which was induced by EGF in the CD24+/CD29hi/DC44hi cell subpopulation. Furthermore, they demonstrated that the HIST2H2AC mRNA was increased by MEK1/2 or PI3-K activation in HC11 and EpH4 mammary epithelial cells, as well as in MC4-L2 and T47-D breast cancer cells. Hist2H2AC silencing was shown to increase EGFR, ERBB2, and ERK1/2 activation and inhibit EGF-induced Zeb-1 expression and E-cadherin down regulation. This study identified an oncogenic role for *HIST2H2AC*, as a regulator of EGF/FGF2 signaling and breast cancer pathophysiology. Their results support a positive feedback loop where EGF stimulates *HIST2H2AC* expression, thereby regulating genes necessary for EGF-induced cell proliferation, apoptosis, EMT, and cell growth.

A study by Bhattacharya *et al.* highlighted the functional significance of HIST1H2AH/HIST1H2AC in regulating Hepatocellular Carcinoma (HCC) pathobiology (42). Mass spectrometry followed by peptide fingerprinting highlighted the differential expression of HIST1H2AH/HIST1H2AC between normal and tumor liver tissues. Furthermore, they highlighted the role of HIST1H2AH/HIST1H2AC in providing a context-dependent growth advantage to the cells, and identified the role of Leu51 and Arg99 residues in conferring a distinct stability to nucleosomes (more detailed discussion below). These results indicate that variable residues in histone isoforms can uniquely regulate an array of biological processes. Hence, the amino acid changes in histone isoforms are subtle but may carry a potential to confer distinct cellular phenotypes.

### Structural impact of histone isoform sequence variations

**Figure 5.**
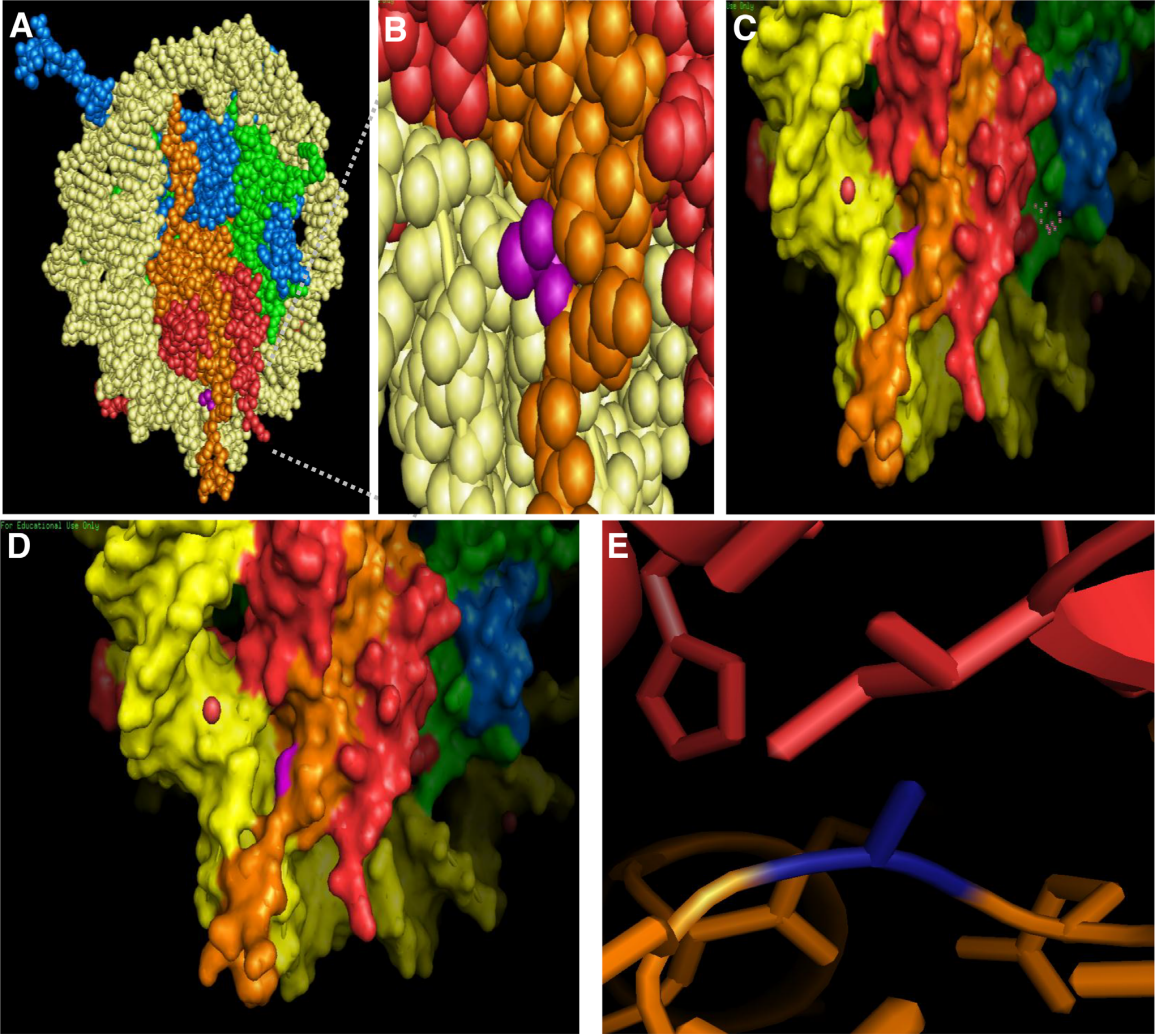
Single amino acid substitutions in H2A affect the nucleosome surface and structure. (**A**) Threonine 16 of H2A is exposed on the surface of the nucleosome. A space-filling model of the canonical nucleosome was generated in PyMOL using the spheres feature. Threonine 16 on H2A is highlighted in purple. (**B**) Zoomed-in view of the region highlighted with a gray box in (A). (**C**) Zoomed-in molecular surface representations of nucleosomes containing threonine **(C)** or serine (**D**) at residue 16 of H2A (highlighted in purple) were generated in pymol using the mutagenesis and surface features. (**E**) Alanine 40 of H2A, highlighted in royal blue, interacts with isoleucine of H2B via hydrophobic side chain interactions. For all images, H3 is light blue, H4 is green, H2A is orange, and H2B is red. The crystal structure of the canonical nucleosome (PDB: 1AOI) was used for all images.

Variations in histone sequence result in alternative nucleosome conformations leading to profound functional implications (55–57). These variations may also affect the accessibility of the underlying DNA leading to alteration of global gene expression patterns (42). Therefore, even minor variations in histone sequence can have substantial effects on cellular physiology by modulating chromatin dynamics. Potential consequences of some of these changes in histone isoform sequences are discussed below:

A recent study by Bhattacharya *et al.* utilized site-directed mutagenesis of specific histone isoforms to identify the critical primary sequence alteration(s) that regulate chromatin dynamics (42). They specifically focused on residues 16, 51 and 99 as these are sites that differ from canonical H2A in H2A1H/H2A1C. Their analysis highlighted that R99K substitution of H2A1H/H2A1C, which is involved in the interaction with the H4 tails in the nucleosome core particle, independently brought about the most significant increase in the dynamics of H2A1H followed by L51M. These dynamic changes include formation of a higher number of H-bonds and multidirectional interactions with proteins and water due to the presence of the guanidinium group in arginine. L51M substitution may also affect nucleosome stability owing to a more favorable solvent transfer term, reduced entropic cost of holding the leucine side chain in a defined position, and increased number of hydrophobic interactions. Additionally, the 51st and the 99th residues are major contributors to the formation of hydrogen bonds with relatively less participation from the 16th residue (42). Furthermore, six of the histone H2A isoforms, including HIST1H2AC, share a substitution of threonine 16 with a serine residue (Figure 1). Threonine 16 is located in the αN-helix of H2A and is surface-exposed in the context of the nucleosome (Figure 5A, B). The loss of a side-chain methyl group from substitution of threonine with serine results in a larger surface-exposed gap between H2A and the DNA (Figure 5C, D). This slight alteration in the nucleosome could potentially affect binding of proteins that recognize nucleosomal surface features. Furthermore, since the αN helix of H2A stabilizes the αC helix of H2B in the nucleosome, and additionally, is involved in hydrogen bonding to DNA, the amino acid substitutions in this region can affect the stability of the H2A–H2B dimer (58).

Another common change is alanine 40 of H2A, located in the L1 loop, which may directly interact with Isoleucine 87 of H2B in the canonical nucleosome (Figure 5C). Our modeling shows that the hydrophobic side chains of alanine and isoleucine interact via van der waals interactions (Figure 5E), which likely results in tighter binding of H2A to H2B and overall a more rigid nucleosome structure. In four of the H2A isoforms, alanine is replaced with a serine at residue 40. The polar side chain of serine could disrupt the hydrophobic interaction with isoleucine, and destabilize the H2A/H2B interaction, altering the biophysical properties of the nucleosome. Furthermore, a mammalian specific O-GlcNAcylation of this residue has been discovered (59). These examples further substantiate that small primary sequence alterations in the nucleosome core can regulate the attainment of distinct physiological states, which may increase the complexity of the epigenome leading to specialized phenotypic traits.

**Figure 6.**
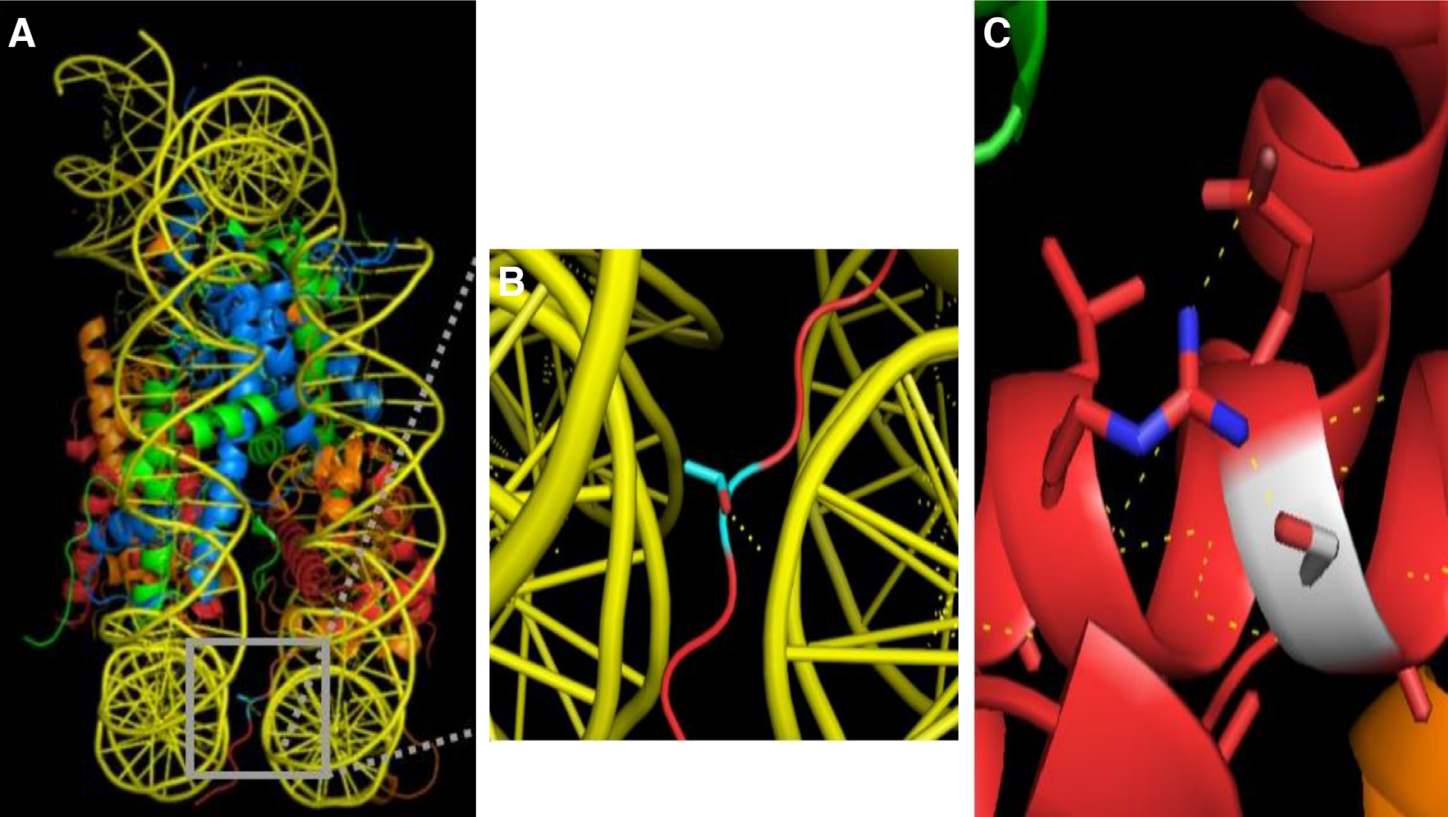
Single amino acid substitutions in H2B affect nucleosome structure. (**A**) Threonine 33 of the H2B N-terminal tail (colored in cyan) is positioned between the DNA gyres of the nucleosome and hydrogen bonds to one strand of DNA. (**B**) Zoomed-in view of the region highlighted with a gray box in (A). Dashed yellow lines indicate hydrogen bonds identified by the PyMol polar contacts tool. (**C**) Serine 76 of H2B (shown in white) makes intra-chain interactions with the adjacent arginine. The hydrogen bond network thus formed between adjacent residues is shown with yellow dashed lines. Glycine 76 was replaced with serine using the PyMol mutagenesis tool. For all images, H3 is light blue, H4 is green, H2A is orange, and H2B is red. The crystal structure of the canonical nucleosome (PDB: 1AOI) was used for all images.

In analogy to H2A, the H2B isoforms also show diversity in terms of their primary sequence (Figure 2). One of the notable variations is the serine/threonine/glycine at position 33 of the H2B isoforms. Threonine 33 within the N-terminal tail of Hist1H2BA is located between two gyres of DNA in the nucleosome. The threonine side chain hydrogen bonds to the DNA (Figure 6A, B), affecting the positioning of the H2B tail that protrudes from the nucleosome. While a serine at position 33 would result in a comparable interaction with the DNA, a glycine at position 33 in HIST3H2BB would result in loss of a hydrogen bond and weaken the interaction of the H2B tail with the nucleosomal DNA. This could potentially result in alternative positioning of the H2B tail outside of the nucleosome, which may affect higher order chromatin structure. Another common alteration involves the substitution of glycine 76 of canonical H2B with a serine in Hist1H2BA, Hist1H2BL and Hist3H2BB isoforms. The polar serine side chain interacts with arginine 80 and results in additional intra-chain interactions within the α2 helix of H2B (Figure 6C). This interaction may result in a more rigid H2B structure affecting the overall nucleosome dynamics.

Among the histone H3 group, the canonical histones including H3.1 and H3.2 vary by a single S96C substitution. This substitution could trigger changes in molecular properties of H3.1, which makes it distinguishable from H3.2, as evident by a specific elution profile using HPLC. A possible explanation may be that this residue is a site for post-translational modification, and therefore, could alter the structure and/or dynamics (60–62). Another study has shown that this substitution can significantly increase the stability of the tetramer. This residue is located within a hydrophobic pocket encompassing F67, A95, and L100 in H3 and L58, F61 and L62 in helix 2 of H4. The presence of cysteine enhances the stability of this cage and subsequently leads to the stabilization of the H3/H4 dimer (63).

H3.1T, the testis specific isoform differs from H3.1 and H3.2 by four substitutions, including A24V, V71M, A98S and A111V (Figure 3). All these modifications of H3.1T confer distinct properties to this isoform, including its *in vitro* and *in vivo* instability, weaker association to H2A/H2B dimer, defective incorporation into the nucleosome by Nap1, and more rapid exchange in the nucleosomes of living cell (64,65).

In the histone H4 group, the Hist1H4G differs from the other two isoforms with 15 substitutions including V2G, A6G, C17R, S23R, C32P, T33A, H40R, L47S, R56G, F58L, W67R, Y68D, N72Y, A85D, V89A, and a deletion of the region spanning from Y98 to 102G. Hist1H4A and Hist1H4I differ by a single change at V70A residue (Figure 4). This residue is located in the α2 helix of histone H4, which is important for interaction with specific chaperones including DAXX and Scm3 (66,67). Furthermore, this residue is critical in generating a hydrophobic pocket, which can adopt different conformations based on physiological changes, thus regulating the interaction with other histones, ubiquitin-binding proteins and DNA repair machinery (68–70). Hence, single amino acid substitutions do carry a potential to alter the cellular environment. Given the abundance of these isoforms, the overall consequences for chromatin structure and its interactome can be substantial.

### Histone isoforms and cancer

While the level of many of the histone isoforms has been known to be altered in the malignant state, their functional significance was not realized, until recently (40,41,71–73). The levels of Hist1H2AC specifically have been reported to change in many diseases including human papillomavirus hyperplasias, AIDS, multiple sclerosis and cancer (74–76). Specifically, Hist1H2AC has been found to be overexpressed in Chronic Myeloid Leukemia (chronic phase), Hairy Cell Leukemia and Hepatocellular Carcinoma and downregulated in Acute Myeloid Leukemia, Colorectal Carcinoma, Esophageal Adenocarcinoma, Hepatocellular Carcinoma and Pancreatic Adenocarcinoma (71). Intriguingly, Hist1H2AC was shown to display a consistent trend of downregulation from normal to low-grade neoplasia (CIN1) to high-grade neoplasia (CIN2 and CIN3), and then to invasive carcinoma (77). Other researchers have found a decrease in the Hist1H2AC levels in MCF-7 cells treated with estradiol (a potent mitogen) and quercetin (78,79). In one study, treatment of HCT116 cells with Nutlin-3a, the levels of the tumor-suppressor p53 increases, with a concomitant decrease in Hist1H2AC levels (80,81). Additionally, the levels of Hist1H2AC have also been shown to decline following a cell cycle arrest (82). Taken together, these studies indicate that the levels of Hist1H2AC in the cell may contribute to the control of proliferation rate and/or tumor suppression.

The abundance of other histone isoforms has also been correlated to multiple stages of carcinogenesis. The level of many H2B isoforms, including Hist1H2BN, has been shown to fluctuate in various malignancies including Nasopharyngeal Carcinoma, Acute Lymphoblastic Leukemia and ovarian cancer (83–85). Additionally, alterations in histone isoform genes have been found to concur with various cancer-driving mutations (86,87). Histone isoforms may also regulation other aspects of cancer pathophysiology. Hist1H2BK is one of the histone isoforms that has shown a direct correlation to metastasis and is known to be elevated in highly metastatic cell lines and stem-like cells (43,88–90). In breast cancer MDA-MB-231 cell line overexpressing different vascular endothelial growth factor (VEGF) isoforms, Hist1H2BK expression displayed a direct correlation with highly metastatic VEGF165 overexpressing cells and statistically negative correlation with less metastatic VEGF189 isoform (89). HIST2H2BC has been shown to correlate with cell proliferation and invasion and contribute to paclitaxel resistance in triple-negative breast cancer cells (91). Similarly, Hist2H2BE has been shown to be altered with angiogenesis, cell proliferation, invasion, apoptosis, and epigenetic therapy (92–95). Hist1H2BL was found to be downregulated in the gemcitabine-resistant pancreatic cancer cell line SW1990/GZ cells compared with control SW1990 cells (96). Intriguingly, Hist3H2BB is also known to be altered by therapies that affect production of reactive oxygen species (ROS), apoptosis, cell proliferation and macromolecular biosynthesis (97,98). The accumulation of these correlations suggests that histone isoforms may play a vital role in the regulation of various ‘hallmarks’ of cancer and therapeutic response, and therefore, invite further exploration. The complete list of replication-dependent histone isoforms and their associated role in carcinogenesis are summarized in Tables 1–4.

**Table 1.** Histone H2A isoforms with the corresponding protein name, molecular weight and relation to carcinogenesis

Gene name(s)	Protein name	Molecular weight	Significance	Reference(s)
HIST1H2AH	H2A 1H	13 817	Negatively regulated by ERβ1, differentially regulated between progressive and non-progressive T1G3 bladder tumors	(99,100)
HIST1H2AJ	H2A 1J	13 847	Correlation to metastasis; overexpressed in aromatase inhibitor resistant estrogen receptor-positive (ER+) breast cancer; specific methylation could result in cell proliferation and anti-apoptosis in HeLa cells	(49,90,101)
HIST2H2AC	H2A 2C	13 899	Regulates proliferation and EMT; highly expressed at the cancer stem-like stage; correlation to metastasis; downregulated in chronic myelogenous leukemia stem cells	(43,90,102–104)
HIST2H2AB	H2A 2B	13 906	Deleted in colon cancer	(105)
HIST1H2AG/I/K/L/M	H2A	14 002		
HIST2H2AA	H2A 2A	14 006	Correlation to metastasis; required for growth and migratory capacity of the photodynamic therapy surviving cells	(103,106)
HIST1H2AC	H2A 1C	14 016	Correlation to metastasis and proliferation; hypoxia induction; migration of ovarian cancer cells; altered in response to epigenetic therapy	(41,106–110)
HIST1H2AD	H2A 1D	14 018	Negatively regulated by ERβ1; increase in acetyl lysine-specific enrichment upon treatment of MDA-MB-231 cells with SAHA; indicator of recurrent carcinomatosis; increasingly expressed in stage IV GC patients	(99,111–113)
HIST3H2A	H2A/3	14 032	Elevated methylation levels in gastric cancer; positive correlation to chemosensitivity in gastric cancer; downregulated in chronic myelogenous leukemia stem cells	(102,114,115)
HIST1H2AB/E	H2A 1B/1E	14 046	Negatively regulated by ERβ1; overexpressed in aromatase inhibitor resistant estrogen receptor-positive (ER+) breast cancer; upregulated after genistein treatment; associated with cycling hypoxia; inversely correlated to metastasis in lung adenocarcinoma; upregulated in head and neck cancer cell lines; downregulated in chronic myelogenous leukemia stem cells; upregulated in T-cell prolymphocytic leukemia	(91,99,101,102, 107,116–119)
HIST1H2AA	H2A 1A	14 102	Altered in androgen-responsive prostate cancer; inversely correlated to metastasis in lung adenocarcinoma	(117,120)

**Table 2.** Histone H2B isoforms with the corresponding protein name, molecular weight and relation to carcinogenesis

Gene name(s)	Protein name	Molecular weight	Significance	Reference(s)
HIST1H2BC	H2B 1C	13 745	Associated with viability of colorectal cancer cells; downregulated in endometrioid carcinoma; overexpressed in metastatic relapse in node-positive breast cancer; altered in response to epigenetic therapy	(88,109,121,122)
HIST1H2BK	H2B 1K	13 801	Correlation with metastasis; Overexpressed in aromatase inhibitor resistant estrogen receptor-positive (ER+) breast cancer; overexpressed in metastatic relapse in node-positive breast cancer; upregulated in dormant breast cancer disseminated tumor cells	(53,88,90,101,103,123)
HIST1H2BH	H2B 1H	13 803	Hypoxia induction; altered in response to proapoptotic and antiangiogenic drugs	(108,124)
HIST1H2BJ	H2B 1J	13 815	Correlation with gastric cancer aggressiveness; downregulated in chronic myelogenous leukemia stem cells	(102,125)
HIST1H2BE/F/G/I/O	H2B	13 817		
HIST3H2BB	H2B 3B	13 819	Elevated methylation levels in gastric cancer, Correlation with gastric cancer aggressiveness; upregulated in head and neck cancer cell lines	(115,119,125)
HIST2H2BE	H2B 2E	13 831	Correlation with gastric cancer aggressiveness; upregulated in breast cancer side population; downregulated in Epithelium from ER+ Breast Cancers compared to reduction mammoplasty controls	(125–127)
HIST1H2BN	H2B 1N	13 833	Upregulated after genistein treatment; downregulated in endometrioid carcinoma, hypomethylated in ovarian tumor-initiating cells	(118,122,128)
HIST1H2BD	H2B 1D	13 847	Inversely correlated to metastasis; associated with viability of colorectal cancer cells; migration of ovarian cancer cells; hypoxia induction; proliferation of bicalutamide-resistant prostate cancer cells; upregulated after caffeic acid phenethyl ester treatment (antioxidant); altered in response to epigenetic therapy	(103,108–110,121,129, 130)
HIST1H2BB	H2B 1B	13 861	Expressed specifically in malignant pool in ovarian cancer tumor fluid; overexpressed in aromatase inhibitor resistant estrogen receptor-positive (ER+) breast cancer; upregulated in head and neck cancer cell lines; downregulated in chronic myelogenous leukemia stem cells	(101,102,119,131)
HIST1H2BL	H2B 1L	13 863	Overexpressed in aromatase inhibitor resistant estrogen receptor-positive (ER+) breast cancer; reduced in patients with severe hemoglobin reduction after S-1 monotherapy in gastric cancer; differentially expressed between vincristine-resistant and control gastric cancer cell lines	(101,132,133)
HIST1H2BM	H2B 1M	13 900	Overexpressed in aromatase inhibitor resistant estrogen receptor-positive (ER+) breast cancer; deleted in colon cancer; expression progressively decreased across breast cancer progression model; expression pattern can distinguish the gastric cancer grades and the control; regulation of apoptosis/cell proliferation	(101,105,134–138)
HIST1H2BA	H2B 1A	14 079	Mutated in neuroendocrine tumors	(139)
HIST2H2BA	H2B 2A	N/A	Altered by interaction of mesenchymal stromal cell with cancer-cell derived extracellular membrane vesicles; differentially regulated between invasive lobular carcinomas and invasive ductal carcinomas of the breast	(140,141)
HIST2H2BB	H2B 2B	N/A	Altered in transgenic mice bladder gene expression for belinostat-treated versus control	(142)
HIST2H2BC	H2B 2C	N/A	Regulation of cell proliferation, invasion and paclitaxel resistance in Triple-negative breast cancer cells	(91)
HIST2H2BD	H2B 2D	N/A	Altered in response to epigenetic therapy	(109)
HIST3H2BA	H2B 3A	N/A	Downregulated in chronic myelogenous leukemia stem cells	(102)

**Table 3.** Histone H3 isoforms with the corresponding protein name, molecular weight and relation to carcinogenesis

Gene name(s)	Protein name(s)	Molecular weight	Significance	Reference(s)
HIST2H3A/B/C	H3.2	15 298	Overexpressed in aromatase inhibitor resistant estrogen receptor-positive (ER+) breast cancer; highly expressed at the cancer stem-like stage; upregulated with CUL4A-mediated increase in proliferation and tumorigenicity of breast cancer cells	(101,104, 143)
HIST1H3A/B/C/D/E/F/G/H/I/J	H3.1	15 315	Negatively regulated by ERβ1; highly expressed at the cancer stem-like stage; overexpressed in aromatase inhibitor resistant estrogen, receptor-positive (ER+) breast cancer; differentially regulated in HER2-positive breast cancer; downregulated in endometrioid carcinoma; upregulated in head and neck cancer cell lines; expression pattern can distinguish the gastric cancer grades and the control; Downregulated in chronic myelogenous leukemia stem cells	(99,101,102, 104,119,122, 135,144)
HIST3H3	H3.1T	15 419	Associated with cycling hypoxia	(107)

**Table 4.** Histone H4 isoforms with the corresponding protein name, molecular weight and relation to carcinogenesis

Gene name(s)	Protein name(s)	Molecular weight	Significance	Reference(s)
HIST1H4G	H4 1G* (needs validation)	10 920	Downregulated in endometrioid carcinoma; upregulated by HuR silencing in tumorigenic thyroid cell line; upregulated in T-cell prolymphocytic leukemia	(116,122,145)
HIST1H4I	H4 1I	11 250	Expressed specifically in malignant pool in ovarian cancer tumor fluid; deleted in colon cancer, downregulated in chronic myelogenous leukemia stem cells; mutated in acral melanoma; hypermethylated in parathyroid adenomas versus normal parathyroid glands	(102,105,131, 146–148)
HIST1H4A/B/C/D/E/F/H/J/K/L	H4	11 278		
HIST2H4				
HIST4H4				

## CONCLUSIONS

The past several years has witnessed an increasing awareness of the potential for replication-dependent histone isoforms to have unique functional properties. The increased interest is likely due to technical advances in a number of fields, such as liquid chromatography and mass spectrometry that have allowed for the separation, identification and quantification of histone isoforms that have subtle differences in primary sequence. This has led to multiple studies demonstrating that the abundance of histone isoforms can vary significantly under a variety of biological contexts. Given the number of genes encoding histone isoforms and their high degree of nucleotide sequence similarity, genetic dissection of unique roles for these histone isoforms has been difficult. This is likely to change with the advent of Crispr-Cas9-mediated gene editing techniques. This technology will allow for creation and analysis of mutations in specific histone isoforms. Importantly, it will also allow for the epitope tagging of histone isoforms in their endogenous loci, permitting the use of ChIP-Seq to determine whether histone isoforms display unique patterns of genomic localization. Combined with increasingly sophisticated biophysical techniques being developed for the analysis of nucleosome and chromatin structure, the functional and mechanistic characterization of histone isoforms may elucidate a novel level of chromatin structure regulation.
